# A cross-sectional study for estimation of associations between education level and osteoporosis in a Chinese men sample

**DOI:** 10.1186/s12891-015-0839-0

**Published:** 2015-12-09

**Authors:** Cai-Xia Yu, Xiu-Zhen Zhang, Keqin Zhang, Zihui Tang

**Affiliations:** Department of Endocrinology and Metabolism, Shanghai Tongji Hospital, Tongji University School of Medicine, Shanghai, 200065 China

**Keywords:** Education level, Osteoporosis, Chinese men, Association

## Abstract

**Background:**

The main aim of this study was to evaluate the association between education level and osteoporosis (OP) in general Chinese Men.

**Methods:**

We conducted a large-scale, community-based, cross-sectional study to investigate the association by using self-report questionnaire to assess education levels. The data of 1092 men were available for analysis in this study. Multiple regression models controlling for confounding factors to include education level were performed to explore the relationship between education level and OP.

**Results:**

Positive correlations between education level and T-score of quantitative bone ultrasound (QUS-T score) were reported (*β* = 0.108, *P* value < 0.001). Multiple regression analysis indicated that the education level was independently and significantly associated with OP (*P* < 0.1 for all models). The men with lower education level had a higher prevalence of OP.

**Conclusion:**

The education level was independently and significantly associated with OP. The prevalence of OP was more frequent in Chinese men with lower education level.

**Trial registration:**

ClinicalTrials.gov Identifier: NCT02451397; date of registration: 05/28/2015).

## Background

Osteoporosis (OP) is a skeletal disorder characterized by compromised bone strength, which predisposes the individual to an increased risk of fractures of the hip, spine, and other skeletal sites [1]. It is widely known that OP is, at least partially, a preventable disease [2]. Bone mineral density is now viewed as a predictor of possible problems [3]. The assessment of risk factors and definition of risk profiles are important steps toward the prevention of fractures in the elderly [4]. This is critical, as China is experiencing a growing osteoporosis pandemic, due to a rapidly developing economy and a large, aging population [5].

Some studies have identified that the prevalence of OP and peripheral fractures are influenced by the educational level of the subject [6]. Research has strongly indicated that a higher education level is associated with a higher likelihood of vitamin D supplementation, which would reduce the risk of OP and bone fractures [7]. Previous studies have described the differences in the prevalence of OP among educational classes and the potentially preventative role played by increased formal education. Using the lowest educational level as a reference category, increases in educational status were associated with a significantly reduced risk for OP in these studies [8]. In addition, other studies have indicated that although half of the pre- and post-menopausal women respondents reported having some awareness of OP, their level of knowledge was poor, particularly with regard to the risk factors associated with the condition and its complications [9]. However, women had attained higher levels of education demonstrated a high level of knowledge about osteoporosis. Evidence indicated that not only a higher level of education but also a lower age were associated with higher levels of knowledge about osteoporosis, with no systematic influence of personal experience with the disease [2]. Studies demonstrated that there were complicated relationships among the levels of attitudes, knowledge, osteoporosis behaviors and educational level [10]. Therefore, there has been an ongoing conversation among researchers regarding the association between education level and OP.

Many studies have shown that better education is effective in lowering the risks for a number of chronic diseases, but little information exists as to how this relates specifically to bone health. Of those studies that have been conducted, more have focused on females than on males [11]. The main purpose of this study was to evaluate the extent to which education level was associated with OP in a sample of Chinese men.

## Methods

### Study population

We performed a community-based study for OP using a random sample of the Chinese population. All participants were recruited from rural and urban communities in Shanghai. Participants aged 30–90 years were included in this study. More than 3,000 participants were invited to a screening visit between 2011 and 2014. Written consent was obtained from all patients before the study, which was performed in accordance with the ethical standards in the Declaration of Helsinki, and approved by the Medicine Ethical Committee of the Shanghai Tongji Hosptial. Some participants with chronic diseases and conditions that might potentially affect bone mass, structure, or metabolism were excluded. Briefly, the exclusion criteria were as follows: a history of 1) serious residual effects of cerebral vascular disease; 2) serious chronic renal disease (Glomerular filtration rate < 30 mL/min/1.73 m^2^); 3) serious chronic liver disease or alcoholism; 4) significant chronic lung disease; 5) corticosteroid therapy at pharmacologic levels; 6) evidence of other metabolic or inherited bone disease, such as hyper- or hypoparathyroidism, Paget disease, osteomalacia, or osteogenesis imperfecta; 7) recent (within the past year) major gastrointestinal disease, such as peptic ulcer, malabsorption, chronic ulcerative colitis, regional enteritis, or significant chronic diarrhea; 8) Cushing syndrome; 9) hyperthyroidism; and 10) any neurologic or musculoskeletal condition that would be a non-genetic cause of low bone mass.

### Data collection

All study subjects underwent complete clinical baseline characteristics evaluation, which included a physical examination and response to a structured, nurse-assisted, self-administrated questionnaire to collect information on age, gender, residential region, visit date, family history, lifestyle, dietary habits, physical activity level during leisure time, use of vitamins and medications, smoking, alcohol consumption, and self-reported medical history. Body weight and height were measured according to a standard protocol. Smoking and alcohol consumption were categorized as never, current (smoking or consuming alcohol regularly in the past 6 months), or ever (cessation of smoking or alcohol consumption for more than 6 months). Regular exercise was defined as any kind of physical activity 3 or more times per week. Self-reported medical history was categorized as “no” or “yes.” HTN was defined as blood pressure ≥140/90 mmHg, or a history of hypertension medication. Diabetes mellitus (DM) was defined by oral glucose tolerance test (OGTT) and either HbAlc ≥ 6.5 % or the use of insulin or hypoglycemic medications.

Education level was evaluated by a semi-quantitative education questionnaire (commonly divided into four stages: group 1: primary school, group 2: junior high school, group 3: senior high school, and group 4: college). To determine education level, the participants were asked, “How about your education level?” The possible answers were: “primary school,” “junior high school,” “senior high school,” or “college,” and the answers were taken as a subjective assessment. To answer the question, the participants were required to decide one issues based on their impressions: 1) whether or not the education experience was actually approved by authority.

### The study outcomes

The bone ultrasound value was measured at calcaneus by standardized quantitative ultrasound (QUS, Hologic Inc., Bedford, MA, USA) utilizing T-scores based on WHO criteria, which were obtained from the automated equipment. T-score refers to the ratio between patient’s ultrasound value and that of young adult population of same sex and ethnicity. T-score of > −1 was taken as normal, between −1 and −2.5 osteopenic and < −2.5 as osteoporotic. Daily calibration was performed during the entire study period by a trained technician. The coefficients of variation of the accuracy of the QUS measurement were 0.9 %. The QUS technology is less expensive, portable and also has the advantage of not using ionising radiation, so it is safer than dualenergy X-ray absorptiometry (DEXA).

### Statistical analysis

Continuous variables were analyzed to determine whether they followed normal distributions, using the Kolmogorov-Smirnov Test. Variables that were not normally distributed were log-transformed to approximate a normal distribution for analysis. Results are described as mean ± SD or median, unless stated otherwise. Differences in variables among subjects grouped by education level were determined by one way analysis of variance. Among groups, differences in properties were detected by *χ*^2^ analysis. Univariate regression analysis was performed to determine variables associated with outcomes (T-score or OP), and to estimate confounding factors possibly disturbing the relation of education level to outcomes (T-score or OP). Tests were two-sided, and a *p*-value of < 0.05 was considered significant. Multiple variables regression (MR) was performed to control potential confounding factors and determine the independent contribution of variables to outcomes (T-score or OP). A *p*-value of < 0.1 was considered significant in MR models.

For the associations analysis, three models have been developed. In model 1, education level were categorized by group 1: primary school, group 2: junior high school, group 3: senior high school, and group 4: college. In model 2: education level were categorized by group 1: low education level including stages of primary school and junior high school, group 2: high education level including stages of senior high school and college. In model 3: education level were categorized by group 1: primary school and group 2: high school and college. Results were analyzed using the Statistical Package for Social Sciences for Windows, version 16.0 (SPSS, Chicago, IL, USA). Odds ratios (OR) with 95 % confidence intervals (CI) were calculated for the relative risk of frequency of fish food intake with the outcome of OP.

## Results

### Clinical characteristics of subjects

The clinical baseline characteristics of the 1092 Chinese male subjects are listed in Table 1. In the total sample, the mean age was 64.11 years. An average T-score of −1.23 was reported and the prevalence of OP was 8.79 % in our study sample. The prevalence of HTN, coronary artery disease (CAD), DM, Gout, and Rheumatoid arthritis (RA) were 45.78, 10.29, 9.73, 3.56, and 3.43 %, respectively. The proportions of subjects having current smoking and alcohol habits were 36.39 % and 30.58 %, respectively. There were significant differences in age, smoking habits, exercise and therapy history among groups according to education level (*P* value <0.05 for all). Significant differences in T-Score and the prevalence of OP among the four groups (*P* value =0.002 for T-score and 0.003 for the prevalence of OP).

**Table 1:** The baseline characteristics of participants

Variables	Total sample	Education level				*P* value
		Primary school	Junior high school	Senior high school	College	
Demographic information						
N	1092	112	366	315	299	-
Age	64.11 ± 9.77	71.1 ± 7.91	62.31 ± 10.22	62.15 ± 10.51	65.77 ± 7.27	<0.001
Height	168.16 ± 5.61	164.67 ± 5.65	168.72 ± 4.15	169.33 ± 6.99	169.17 ± 6.34	0.030
Weight	67.96 ± 11.94	65.09 ± 10.25	69.24 ± 13.89	67.87 ± 9.26	66.75 ± 10.82	0.654
Lifestyle						
Smoking	397(36.39 %)	41(36.61 %)	159(43.56 %)	127(40.32 %)	70(23.41 %)	<0.001
Alcohol intake	333(30.58 %)	38(34.23 %)	126(34.52 %)	93(29.62 %)	76(25.42 %)	0.064
Excise	705(64.56 %)	64(57.14 %)	213(58.2 %)	200(63.49 %)	228(76.25 %)	<0.001
Medical history						
HTN	494(45.78 %)	56(50.45 %)	157(43.13 %)	144(46.45 %)	137(46.6 %)	0.544
CAD	108(10.29 %)	11(10 %)	30(8.5 %)	37(12.21 %)	30(10.56 %)	0.480
DM	104(9.73 %)	12(10.81 %)	32(8.91 %)	24(7.84 %)	36(12.29 %)	0.279
RA	37(3.43 %)	5(4.5 %)	9(2.48 %)	11(3.54 %)	12(4.1 %)	0.620
Therapy history						
Vitamin C	115(10.53 %)	5(4.46 %)	22(6.01 %)	33(10.48 %)	55(18.39 %)	<0.001
Vitamin D	29(2.66 %)	0(0 %)	4(1.09 %)	7(2.22 %)	18(6.02 %)	<0.001
Outcomes						
T-score	−1.23 ± 0.91	−1.42 ± 1.06	−1.3 ± 0.86	−1.21 ± 0.85	−1.08 ± 0.95	0.002
OP	96(8.79 %)	20(17.86 %)	29(7.92 %)	20(6.35 %)	27(9.03 %)	0.003

Note: *HTN* hypertension, *CAD* coronary artery disease, *DM* diabetes mellitus, *RA* Rheumatoid arthritis, *OP* Osteoporosis

### Association analysis for T-score

**Fig. 1:**
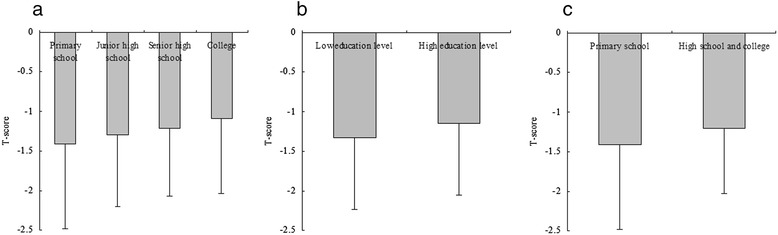
Comparison of T score among groups according to education level. **a**, The results of comparison of T-score among groups according to Model 1 (education level were categorized by group 1: primary school, group 2: junior high school, group 3: senior high school, and group 4: college). The mean T-score was −1.42, –1.30, -1.21 and −1.08 in the four groups, respectively. There were significantly differences among the four groups (*P* value = 0.002). **b**, The results of comparison of T-score among groups according to Model 2 (education level were categorized by group 1: low education level - primary school and junior high school, and group 2: high education level - senior high school and college). The mean T-score was −1.33 and −1.15 in the two groups, respectively. There were significantly differences between the two groups (*P* value =0.001). **c**, The results of comparison of T-score between groups according to Model 3 (education level were categorized by group 1: primary school, and group 2: high school and college). The mean T-score was −1.42 and −1.21 in the two groups, respectively. There were significantly differences between the two groups (*P* value =0.021)

Univariate linear regression analyses were developed to include demographical information, medical history, and lifestyle to estimate the association of various clinical factors and T-score (Table 2). The variables age, exercise, and education level were significantly associated with the T-score. The comparison of T-scores among groups according to education level (group 1: primary school, group 2: junior high school, group 3: senior high school, and group 4: college) reported that the mean T-score was −1.42, −1.30, −1.21 and −1.08 in the four groups, respectively (Fig. 1a). There were significant differences among the four groups (*P* value = 0.002). Additionally, there were significant differences among groups according to model 2 and model 3 (Fig. 1b and 1c, *P* value = 0.001 for model 2 and *P* value = 0.021 for model 3). Univariate linear analysis demonstrated a positive correlation between education level and T-score.

**Table 2:** Univariate linear regression analysis for associations among variables and T-score

Variables	*β*	SE	*P* value	95 % CI for B
Age	−0.009	0.003	0.002	−0.014–0.003
Height	−0.002	0.016	0.911	−0.034–0.030
Weight	0.008	0.008	0.279	−0.007–0.023
HTN	0.087	0.056	0.117	−0.022–0.196
CAD	−0.085	0.092	0.359	−0.266–0.096
DM	0.060	0.095	0.523	−0.125–0.246
RA	−0.248	0.153	0.105	−0.547–0.052
Smoking	−0.044	0.057	0.441	−0.157–0.068
Alcohol intake	−0.048	0.060	0.420	−0.166–0.069
Exercise	0.060	0.024	0.049	1.002–0.121
Vitamin D	0.026	0.172	0.881	−0.311–0.363
Education level	0.108	0.028	<0.001	0.053–0.163

Note: *HTN* hypertension, *CAD* coronary artery disease, *DM* diabetes mellitus, *RA* rheumatoid arthritis

Multivariate linear regression analyses were developed to include education level and the outcome of T-score. After adjustment for relevant potential confounding factors, the multivariate linear regression analyses detected significant associations (*β* = 0.097, *p*-value = 0.001, 95 % CI: 0.039–0.155 for model 1; *β* = 0.162, *p*-value = 0.005, 95 % CI: 0.048–0.277 for model 2). No significant associations were reported in model 3 (*p*-value = 0.299). The data were shown in Table 3.

**Table 3:** Multiple variables linear regression analysis for the associations between education level and T score

Model	Variable	*β*	SE	*P* value	95 % CI for *β*
Model 1	Education level	0.097	0.030	0.001	0.039–0.155
Model 2	Education level	0.162	0.058	0.005	0.048–0.277
Model 3	Education level	0.063	0.061	0.299	−0.056–0.182

Note: Model 1: education level were categorized by group 1: primary school, group 2: junior high school, group 3: senior high school, and group 4: college; Model 2: education level were categorized by group 1: low education level (primary school and junior high school), and group 2: high education level (senior high school and college); Model 3: education level were categorized by group 1: primary school, and group 2: high school and college; and all models were adjusted for age, smoking, alcohol intake, exercise and medical and therapy history

### Association analysis for OP

**Fig. 2:**
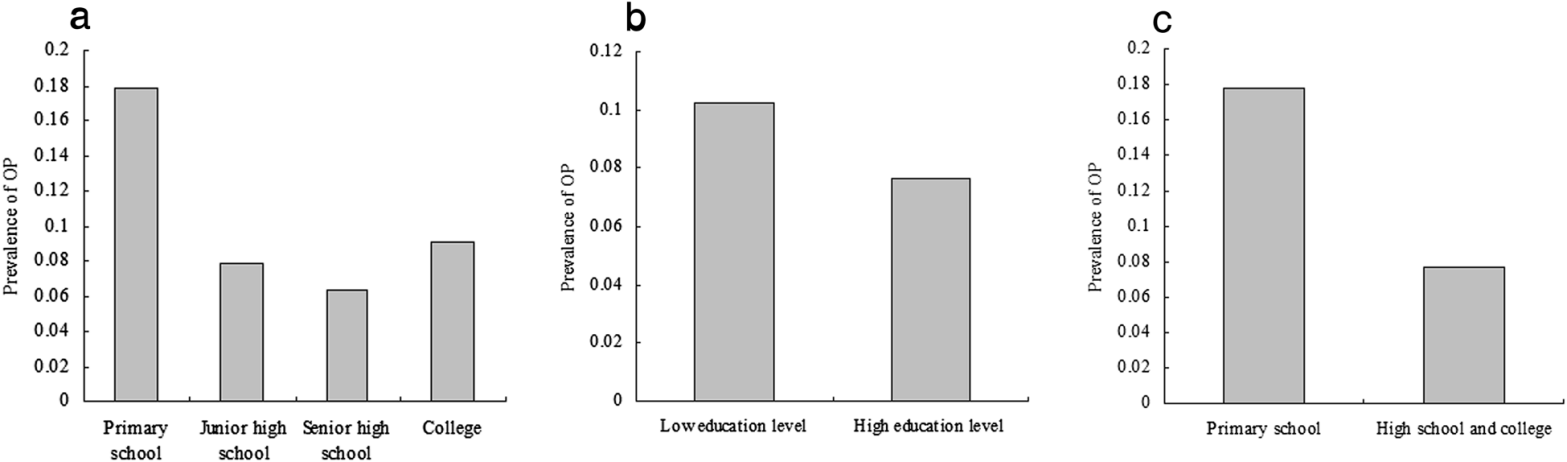
Comparison of prevalence of osteoporosis among groups according to education level. **a**, The results of comparison of prevalence of osteoporosis among groups according to Model 1 (education level were categorized by group 1: primary school, group 2: junior high school, group 3: senior high school, and group 4: college). The prevalence of osteoporosis was 17.86, 7.92, 6.35 and 9.03 % in the four groups, respectively. There were significantly differences among the four groups (*P* value = 0.003). **b**, The results of comparison of prevalence of osteoporosis among groups according to Model 2 (education level were categorized by group 1: low education level - primary school and junior high school, and group 2: high education level - senior high school and college). The prevalence of osteoporosis was 10.25 % and 7.65 % in the two groups, respectively. There were significantly differences between the two groups (*P* value = 0.133). **c**, The results of comparison of prevalence of osteoporosis between groups according to Model 3 (education level were categorized by group 1: primary school, and group 2: high school and college). The prevalence of osteoporosis was 17.86 % and 7.76 % between the two groups, respectively. There were significantly differences between the two groups (*P* value = 0.001)

Univariate logistic analyses were performed to evaluate associations with OP. The results indicate that age, RA, alcohol intake, and exercise were significantly associated with OP (*P* value < 0.05 for all, Table 4). The comparison of prevalence of OP among groups according to model 1 revealed that the prevalence of OP was 17.86, 7.92, 6.35 and 9.03 % in the four groups, respectively (Fig. 2a). There were significant differences among the four groups (*P* value = 0.003). In addition, significant differences among groups according to model 3 were reported (Fig. 2c, *P* value = 0.001 for model 3). Correlation analysis demonstrated a negative correlation between education level and OP (data not shown).

**Table 4:** Univariate logistic regression analysis for associations among variables and osteoporosis

Variable	*β*	S.E.	*P* value	OR	95.0 % CI
Age	0.072	0.013	<0.01	1.074	1.047–1.102
Height	−0.156	0.097	0.065	0.856	0.735–0.996
Weight	−0.052	0.048	0.279	0.950	0.865–1.043
HTN	−0.023	0.216	0.915	0.977	0.640–1.492
CAD	0.388	0.318	0.222	1.474	0.791–2.75
DM	0.097	0.351	0.784	1.101	0.553–2.193
RA	1.107	0.415	0.008	3.025	1.342–6.820
Smoking	−0.245	0.143	0.066	0.783	0.616–1.006
Alcohol intake	−0.344	0.134	0.010	0.709	0.545–0.921
Excise	−0.274	0.137	0.045	0.760	0.582–0.994
Vitamin D	0.800	0.504	0.112	2.225	0.829–5.972
Education level	−0.197	0.11	0.073	0.821	0.662–1.018

Note: *HTN* hypertension, *CAD* coronary artery disease, *DM* diabetes mellitus, *RA* rheumatoid arthritis

Multivariate logistic regression analyses were employed to evaluate the association between education level and the OP outcome. After adjustment for relevant potential confounding factors, the multivariate logistic regression analysis detected significant associations (*p*-value = 0.036 for model 1, *p*-value = 0.096 for model 2, and *p*-value = 0.005 for model 3, Table 5). In participants with education level of primary school, the OR for OP was 2.28 in model 3.

**Table 5:** multiple variables logistic regression analysis for associations between education level and osteoporosis

Model	Variable	*β*	S.E.	*P* value	OR	95 % CI
Model 1	Education level	−0.240	0.114	0.036	0.787	0.629–0.985
Model 2	Education level	−0.387	0.233	0.096	0.679	0.430–1.072
Model 3	Education level	−0.825	0.295	0.005	0.438	0.246–0.782

Note: Model 1: education level were categorized by group 1: primary school, group 2: junior high school, group 3: senior high school, and group 4: college; Model 2: education level were categorized by group 1: low education level (primary school and junior high school), and group 2: high education level (senior high school and college); Model 3: education level were categorized by group 1: primary school, and group 2: high school and college; and all models were adjusted for age, smoking, alcohol intake, exercise and medical and therapy history

## Discussion

A large-scale, community-based, cross-sectional study was conducted to estimate the association between education levels and prevalence of OP among a sample of Chinese men. The prevalence of common diseases, such as DM, CAD, and HTN, was consistent with the results of nationwide epidemiological studies, and our sample was determined to be an adequate representation of the Chinese male population. A subjective self-reported questionnaire estimating education level was suitable for this large-scale study because of its convenience in collecting independent variables. Importantly, our study is the first performed on a large sample of Chinese males in order to obtain data about the relationship between education levels and OP. The answers to the questionnaire were collected during the years 2011–2015. In this study, T score was evaluated using QUS, which has many advantages in assessing osteoporosis: the modality is small, no ionizing radiation is involved, measurements can be made quickly and easily, and the cost of the device is low compared with DXA and quantitative computed tomography devices.

The primary finding of our study was that education level was strongly, independently, and significantly associated with OP in Chinese men. Results of univariate and multiple variable analysis provided evidence to support our findings (*p*-value <0.05 for all analyses). Specifically, in individuals who had attained a low level of education, the OR was >1.0, indicating that the prevalence of OP was higher in these individuals. These results were consistent with those of other studies in which low education level was linked to lower BMD and OP. For example, Etemadifar et al. conducted a population-based, cross-sectional study to explore the relationship between education level and knowledge about OP in a sample of healthy Iranian women, suggesting that subjects with a higher education level possessed significantly better knowledge about OP than did women with a lower educational level [12]. A high level of education and having OP or knowing somebody with OP were the strongest predictive factors for knowledge about this disease in the Norwegian study [13]. Kim et al. have indicated that after adjusting for age, sex, and health behaviors, level of education was only significantly associated with osteoporosis in men, whereas education level had an inverse relationship with osteoporosis only in women (*p* = 0.01, *p* < 0.001, respectively) [14]. Education level was associated with OP, and individuals who had attained a low level of education were linked to high prevalence of OP. Our finding provided strong evidence that education level has a positive correlation and association with OP in Chinese men.

Some authors have emphasized that a subject’s level of knowledge regarding OP is associated with his or her level of education [15]. Education level was positive correlation with knowledge. The participants with high education level have a more knowledge of prevention and management of this disease. For example, Drozdzowska et al. reported that subjects with a higher level of education offered correct answers more frequently. The data they collected provided important information about knowledge of OP among their sample. Our study and other epidemiological studies demonstrated that education level is positively correlated with OP. The independent and significant association between education level and BMD has been identified and explained by several medical studies. In this study, we did not take into account that education level may represent a greater contribution to OP than some other factors.

The current study has several limitations. Firstly, it does not cover age groups outside of 30–90 years. Additionally, all subjects were recruited from an urban, industrial region, and comparison with a rural population is not possible. The study data, based on a cross-sectional study for association analysis, also requires a larger sample size and more geographic representations. Thirdly, a subjective self-reported questionnaire was used to estimate education level for convenience in a large-scale cross-sectional study. Our sample was sufficiently large to account for statistically significant considerations and consisted of men who fell along a wide spectrum of age and educational levels. Finally, it is important to mention that our study was conducted in Chinese men, and our findings may not be relevant for application to other ethnicities.

## Conclusion

Our findings indicated that education level was independently and significantly associated with OP. Specifically, the prevalence of OP was more frequent in Chinese men who had attained a low level of education. This study suggested that improvement in access to education might be beneficial in the prevention of OP in Chinese men.

## Ethical approval

All procedures performed in studies involving human participants were in accordance with the ethical standards of the institutional and/or national research committee and with the 1964 Helsinki declaration and its later amendments or comparable ethical standards.

## Abbreviations

BMD: bone mineral density
BMI: body mass index
BM-MNC: bone marrow-derived mononuclear cell
CAD: coronary artery disease
CI: confidence interval
DM: diabetes mellitus
DXA: dual-energy X-ray absorptiometry
GFR: glomerular filtration rate
HTN: hypertension
MR: multiple variable regression
OP: osteoporosis
OR: odds ratio
QUS: quantitative ultrasound
RA: rheumatoid arthritis

## References

[1] Lane NE (2006). Epidemiology, etiology, and diagnosis of osteoporosis. Am J Obstet Gynecol.

[2] Drozdzowska B, Pluskiewicz W, Skiba M (2004). Knowledge about osteoporosis in a cohort of Polish females: the influence of age, level of education and personal experiences. Osteoporos Int.

[3] Goddard D, Kleerekoper M (1998). The epidemiology of osteoporosis. Practical implications for patient care. Postgrad Med.

[4] Lips P (1997). Epidemiology and predictors of fractures associated with osteoporosis. Am J Med.

[5] Zhang Z, Ho S, Chen Z, Zhang C, Chen Y (2014). Reference values of bone mineral density and prevalence of osteoporosis in Chinese adults. Osteoporos Int.

[6] Allali F, Rostom S, Bennani L, Abouqal R, Hajjaj-Hassouni N (2010). Educational level and osteoporosis risk in postmenopausal Moroccan women: a classification tree analysis. Clin Rheumatol.

[7] Castro-Lionard K, Dargent-Molina P, Fermanian C, Gonthier R, Cassou B (2013). Use of calcium supplements, vitamin D supplements and specific osteoporosis drugs among french women aged 75–85 years: patterns of use and associated factors. Drugs Aging..

[8] Varenna M, Binelli L, Zucchi F, Ghiringhelli D, Gallazzi M (1999). Prevalence of osteoporosis by educational level in a cohort of postmenopausal women. Osteoporos Int.

[9] Okumus M, Ceceli E, Tasbas O, Kocaoglu S, Akdogan S (2013). Educational status and knowledge level of pre-and postmenopausal women about osteoporosis and risk factors: A cross-sectional study in a group of Turkish female subjects. J Back Musculoskelet Rehabil.

[10] Puttapitakpong P, Chaikittisilpa S, Panyakhamlerd K, Nimnuan C, Jaisamrarn U (2014). Inter-correlation of knowledge, attitude, and osteoporosis preventive behaviors in women around the age of peak bone mass. BMC Womens Health.

[11] Ho SC, Y-m C, Woo JL (2005). Educational level and osteoporosis risk in postmenopausal Chinese women. Am J Epidemiol.

[12] Etemadifar MR, Nourian S-M, Fereidan-Esfahani M, Shemshaki H, Nourbakhsh M (2013). Relationship of knowledge about osteoporosis with education level and life habits. World Journal of Orthopedics..

[13] Magnus J, Joakimsen R, Berntsen G, Tollan A, Søgaard A (1996). What do Norwegian women and men know about osteoporosis?. Osteoporos Int.

[14] Kim J, Lee J, Shin J-Y, Park B-J (2015). Socioeconomic disparities in osteoporosis prevalence: different results in the overall Korean adult population and single-person households. J Prev Med Public Health.

[15] Hamid S, Al-Ghufli FR, Raeesi HA, Al-Dhufairi KM, Al-Dhaheri NS (2014). Women’s knowledge, attitude and practice towards menopause and hormone replacement therapy: a facility based study in Alain, United Arab Emirates. J Ayub Med Coll Abbottabad.

